# Skeletal remains of a Pleistocene modern human *(Homo sapiens)* from Sulawesi

**DOI:** 10.1371/journal.pone.0257273

**Published:** 2021-09-29

**Authors:** Adam Brumm, David Bulbeck, Budianto Hakim, Basran Burhan, Adhi Agus Oktaviana, Iwan Sumantri, Jian-xin Zhao, Maxime Aubert, Ratno Sardi, David McGahan, Andi Muhammad Saiful, Shinatria Adhityatama, Yousuke Kaifu

**Affiliations:** 1 Australian Research Centre for Human Evolution, Griffith University, Brisbane, Australia; 2 Archaeology and Natural History, School of Culture, History and Language, College of Asia and the Pacific, Australian National University, Canberra, Australia; 3 Balai Arkeologi Sulawesi Selatan, Makassar, Indonesia; 4 Pusat Penelitian Arkeologi Nasional (ARKENAS), Jakarta, Indonesia; 5 Place, Evolution and Rock Art Heritage Unit, Griffith Centre for Social and Cultural Research, Griffith University, Gold Coast, Australia; 6 Archaeology Laboratory, Hasanuddin University, Makassar, Indonesia; 7 School of Earth & Environmental Sciences, University of Queensland, St. Lucia, Queensland, Australia; 8 The University Museum, The University of Tokyo, Bunkyo, Tokyo, Japan; University of Florence, ITALY

## Abstract

Major gaps remain in our knowledge of the early history of *Homo sapiens* in Wallacea. By 70–60 thousand years ago (ka), modern humans appear to have entered this distinct biogeographical zone between continental Asia and Australia. Despite this, there are relatively few Late Pleistocene sites attributed to our species in Wallacea. *H*. *sapiens* fossil remains are also rare. Previously, only one island in Wallacea (Alor in the southeastern part of the archipelago) had yielded skeletal evidence for pre-Holocene modern humans. Here we report on the first Pleistocene human skeletal remains from the largest Wallacean island, Sulawesi. The recovered elements consist of a nearly complete palate and frontal process of a modern human right maxilla excavated from Leang Bulu Bettue in the southwestern peninsula of the island. Dated by several different methods to between 25 and 16 ka, the maxilla belongs to an elderly individual of unknown age and sex, with small teeth (only M^1^ to M^3^ are extant) that exhibit severe occlusal wear and related dental pathologies. The dental wear pattern is unusual. This fragmentary specimen, though largely undiagnostic with regards to morphological affinity, provides the only direct insight we currently have from the fossil record into the identity of the Late Pleistocene people of Sulawesi.

## Introduction

**Fig 1:**
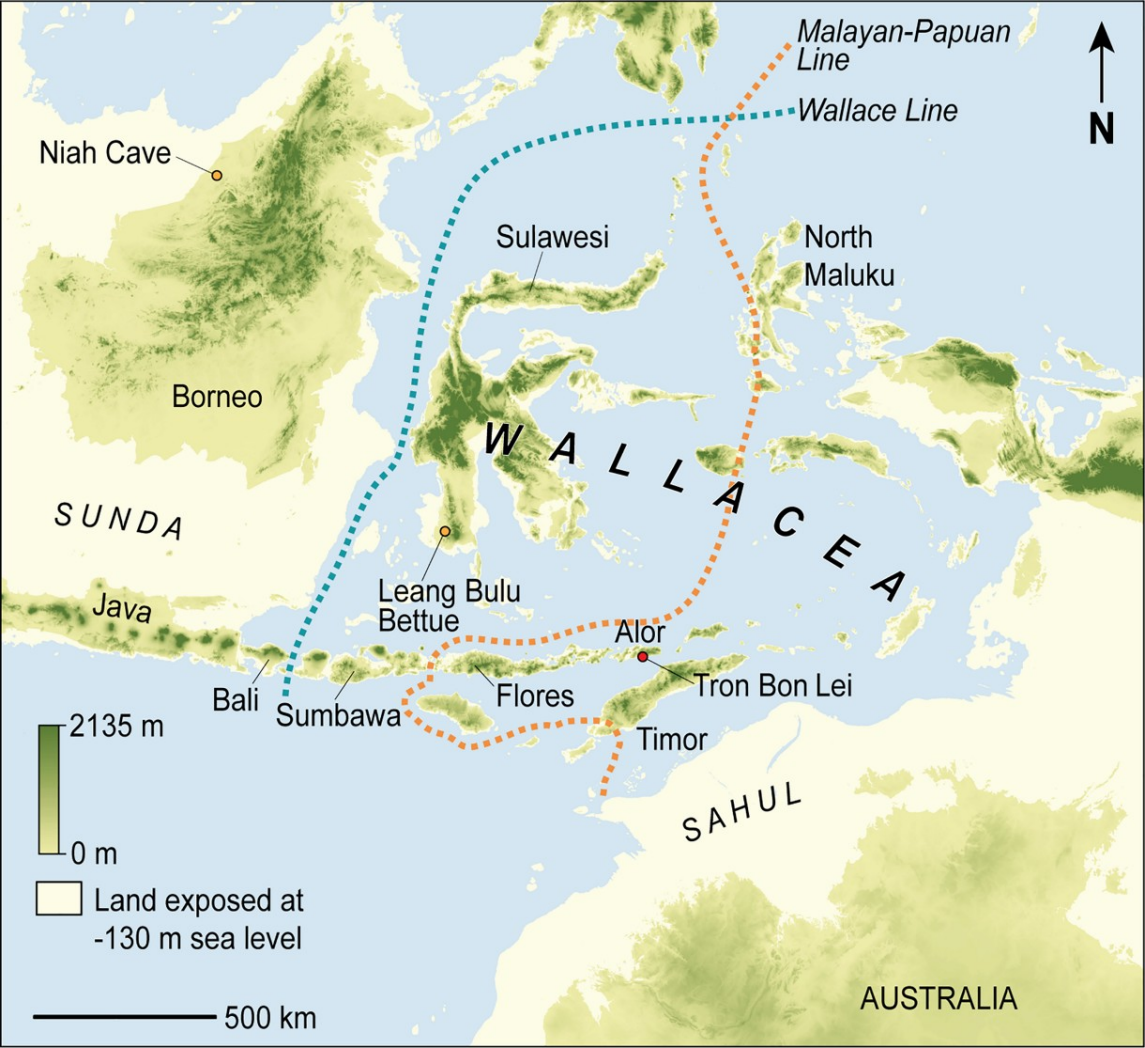
Map of Wallacea showing the location of Sulawesi. The Late Pleistocene cave site Leang Bulu Bettue is located in the island’s southwestern peninsula, known as South Sulawesi. Wallacea comprises an extensive zone of oceanic islands located east of a significant biogeographical boundary, the Wallace Line. This archipelago is positioned between the continental regions of Asia (Sunda) and Australia-New Guinea (Sahul). The Malayan-Papuan Line delineates a major east-west division in the genetic diversity of modern human populations in Wallacea. Map source, Shuttle Radar Topography Mission 1 Arc-Second Global by NASA/NGS/USGS; GEBCO_2014 Grid, version 20150318 (http://gebco.net). Base map generated using ArcGIS by M. Kottermair and A. Jalandoni.

The skeletal remains of anatomically modern humans (AMH) are rare in the Late Pleistocene record of Island Southeast Asia. The evidence at hand is currently limited to a small number of specimens excavated from Borneo, Java, Palawan, and Alor [1,2]. AMH remains are especially scarce in the Wallacean archipelago, or Wallacea [2], a biogeographically distinct zone comprised of thousands of oceanic islands (Fig 1). Wallacea lies between the edge of the Southeast Asian continental shelf (Sunda) and the ‘super-continent’ of Sahul, the landmass that emerged during the Pleistocene at times when global sea levels receded far enough to drain the shallow sea strait dividing mainland Australia from New Guinea. None of the ~2000 islands in Wallacea have ever been connected to Sunda or Sahul, even at the height of the Last Glacial Maximum (LGM; 22–19 ka) when global sea levels reduced by up to 130 m.

Wallacea has a long and enigmatic history of occupation by AMH. Current evidence suggests that the initial peopling of northern Sahul had taken place by 50 ka, as revealed by excavations at multiple localities across this region [3], and possibly by 70–60 ka, which findings at a single site (Madjedbebe) may indicate [4]. The latter claim remains contentious, however [3]. It has long been plainly evident that the first AMH group(s) to make landfall on Sahul must have colonised at least some parts of Wallacea first. At present, however, it remains uncertain which particular islands were settled by AMH during their earliest movements east of Sunda and which, if any, were left uncolonised. Theoretical debates continue over the precise marine voyaging route(s) used by AMH on their crossing from Sunda to Sahul, with the most widely investigated scenarios revolving around the northern route from Borneo via Sulawesi to the Bird’s Head of New Guinea and the southern route from Bali to Timor and thereafter to Australia [5–9]. However, it is now evident that both routes are theoretically possible. The current level of uncertainty surrounding the earliest movements of our species in the region largely stemming from the lack of sustained research efforts in Wallacea [10,11]. Fieldwork projects focused on the Late Pleistocene period in the region have been increasing in number, scale, and scope over the past 20 years or so, but there are still relatively few well-dated sites from this key phase in the deep human past of Wallacea [10]. Presently, the oldest excavated artefacts attributed to AMH date to around 44.6 thousand calibrated radiocarbon years before present (cal ka BP) at Laili cave in the eastern part of Timor (Timor-Leste) [12], while the earliest proxy evidence for our species comprises a figurative rock painting of an animal dated to at least 45.5 ka at the limestone cave of Leang Tedongnge in southern Sulawesi (see below) [13]. There is therefore a gap of several millennia between the oldest widely accepted sites in Sahul (~50 ka; [3]) and the earliest archaeological evidence attributed to our species in Wallacea.

The modern human skeletal record in Wallacea is particularly meagre [2]. Formerly, the oldest AMH skeletal remains consisted of three relatively complete individuals dating to ~17–12 ka at Tron Bon Lei rock-shelter on the small island (2100 km^2^) of Alor in southeastern Wallacea [2,11] (Fig 1). Alor is adjacent to Timor and may have been one of the ‘last stops’ on the southern route to Sahul. It also lies on the eastern side of a major east-west division in the genetic diversity of modern people in Wallacea, the so-called ‘Malayan-Papuan Line’ (Fig 1) which separates the archipelago into two distinct genetic zones along a boundary running between Flores and Sumbawa in the south and the Malukus in the north [14]. On the western side, Y chromosome and mtDNA haplogroups are East Asian in origin, whereas to the east most people have an ancestral composition dominated by Papuan lineages. This abrupt transition in the genetic ancestry of people living on opposite sides of Wallacea reflects the ‘Neolithic’ settlement history of Austronesian-speaking farming societies from mainland Asia, but may also be partly explained by a much earlier and still poorly understood pattern of Late Pleistocene human migrations, including extensive LGM population movements [14]. Unravelling the origins of the Malayan-Papuan Line requires a far more complete record of Pleistocene *H*. *sapiens* fossils from both sides of this major east-west division in present-day peoples of Wallacea.

A confounding factor is the presence of two now-extinct hominin lineages (and possibly more) in Wallacea at around the time our species is thought to have established itself in the region: *Homo floresiensis* from the Late Pleistocene of Flores (~100–60 ka) [15–17], and *Homo luzonensis* from Callao Cave in the northern Philippines island of Luzon (~67 ka) [18]. In Sulawesi, the earliest archaeological evidence comes from the Walanae Depression in the island’s southwestern peninsula. These findings consist of stone tools excavated from deeply stratified deposits at an open-air site (Talepu) dated to ~194–118 ka [19]. The Talepu stone artefacts are technologically straightforward and are not associated with human fossils (as-yet undiscovered at this site). The identity of the hominins responsible for making them is not known [19]. It has also been hypothesised, based on complex statistical analyses of modern genetic data, that the Denisovans were split into at least two distinct lineages [20], one of which may have been present in Wallacea long before the first AMH arrived [21]. Indeed, it has even been proposed that one of these Denisovan branches reached Sahul (New Guinea) and persisted in this northern part of the continent until as recently as 30–14.5 ka [22]. This idea is contentious; if correct, it would imply that: 1) Denisovans inhabited Sahul at the same time as *H*. *sapiens*, apparently for a significant length of time; and 2) Denisovans were capable of major sea-crossings east of Sunda and thus potentially could have had an extensive geographical spread in Wallacea. Finally, the presence in the region of at least two other early human ‘ghost’ species is also inferred from patterns of archaic introgression in the genomes of modern-day people in various parts of Island Southeast Asia, Melanesia, and the wider region [23–25].

Given the empirical observations gleaned from the fossil record, and the various speculative hypotheses based on genetic data from humans both living and ancient, determining which lithic assemblages and other archaeological materials in Late Pleistocene Wallacea can be attributed to AMH, in the absence of their fossils, is not straightforward. This is especially so for Wallacean islands known or suspected to have been inhabited by archaic hominins close to the time of AMH colonisation.

Here, we report on the discovery of the first human skeletal remains from the Pleistocene of Sulawesi, the largest island in Wallacea and the most significant landmass on the northern route to Sahul and the western side of the Malayan-Papuan Line. Research into the prehistoric archaeology of Sulawesi began in the early twentieth century [26]; in fact, up until recent decades it was the most intensively explored island from an archaeological perspective in Wallacea, and, outside Java, in all of Indonesia. As with most parts of the region, however, research progress in Sulawesi has been slow and sporadic. Indeed, until well into the twenty-first century there had essentially been only two excavated archaeological localities that had produced dated evidence for Late Pleistocene human occupation [27,28]–two sites for an island which, at around 174,000 km^2^, is the world’s eleventh largest. In this paper, we report on our ongoing excavations at the limestone cave of Leang Bulu Bettue in the south of Sulawesi, work that has uncovered a partial AMH maxilla and associated skeletal elements in deposits dating to between around 25 and 16 ka. We describe the context and chronology of the newly uncovered human fossil remains and present a morphological description of these materials.

### Find context

The site of Leang Bulu Bettue is located in the limestone tower karst region of Maros in the southwestern peninsula of Sulawesi (Fig 1). The ~450 km^2^ lowland karsts of Maros and the adjoining Pangkajene (or Pangkep) karsts further north lie between 4°7’ S and 5°1’ S [29]. This extensive karstic landscape harbours hundreds of caves and rock-shelters containing archaeological evidence for prehistoric human habitation, including parietal artworks (rock art). Concerning the latter, Uranium-series (U-series) dating of a coralloid speleothem associated with a hand stencil at Leang Timpuseng in Maros-Pangkep produced a minimum age estimate of 40 ka [30]. It is also evident that early humans continued to produce hand stencils in the karst caves and shelters of Maros-Pangkep until ~27–23 ka, based on bracketing U-series ages obtained from speleothem layers ‘sandwiching’ hand stencil art [30]. Most recently, U-series dating in the lowland karst district of Maros-Pangkep yielded securely dated evidence for what seems to be the world’s earliest known figurative representation of the animal world [13]. This rock art panel portraying Sulawesi warty pigs (*Sus celebensis*) has a minimum age of 45.5 ka, based on U-series dating of an overlying coralloid speleothem [13]. Until recently, the earliest excavated archaeological findings in the Maros-Pangkep karsts dated to 35.6–34.5 cal ka BP, as revealed by Glover’s 1975 excavations at Leang Burung 2 rockshelter in the Leang-Leang valley [28]; but see [31] for a major revision of the archaeological sequence at this well-known prehistoric site]. Some 20 km to the north, in the Pangkep district, excavations at the high-level cave of Leang Sakapao 1 have yielded *in situ* stone artefacts and shellfish remains with a maximum age of 30–20 cal ka BP [27].

Elsewhere, we have published preliminary observations on the archaeological sequence at Leang Bulu Bettue, a new Late Pleistocene human occupation site in the Maros-Pangkep karsts [10,32,33]. Located in the Leang-Leang valley, Leang Bulu Bettue is a limestone cave and rock-shelter positioned at valley-floor level around 20 km from the present shoreline to the west. It has a cave mouth measuring 4 m in width and 3 m in height, and an interior chamber that is 27.3 m long, 12.6 m wide, and up to 9.2 m high. The rockshelter area outside the cave extends for a distance of ~30 m along the base of the overhanging limestone cliff face. The shelter roof is located 15.6 m above the floor. Rock art at the site comprises undated red hand stencils (N = 37), most of which are poorly preserved. Superimposed on these traces of Pleistocene-style rock art are stylistically distinct charcoal drawings (including images of ‘dancing’ anthropomorphic figures) produced during the late Holocene [30].

**Fig 2:**
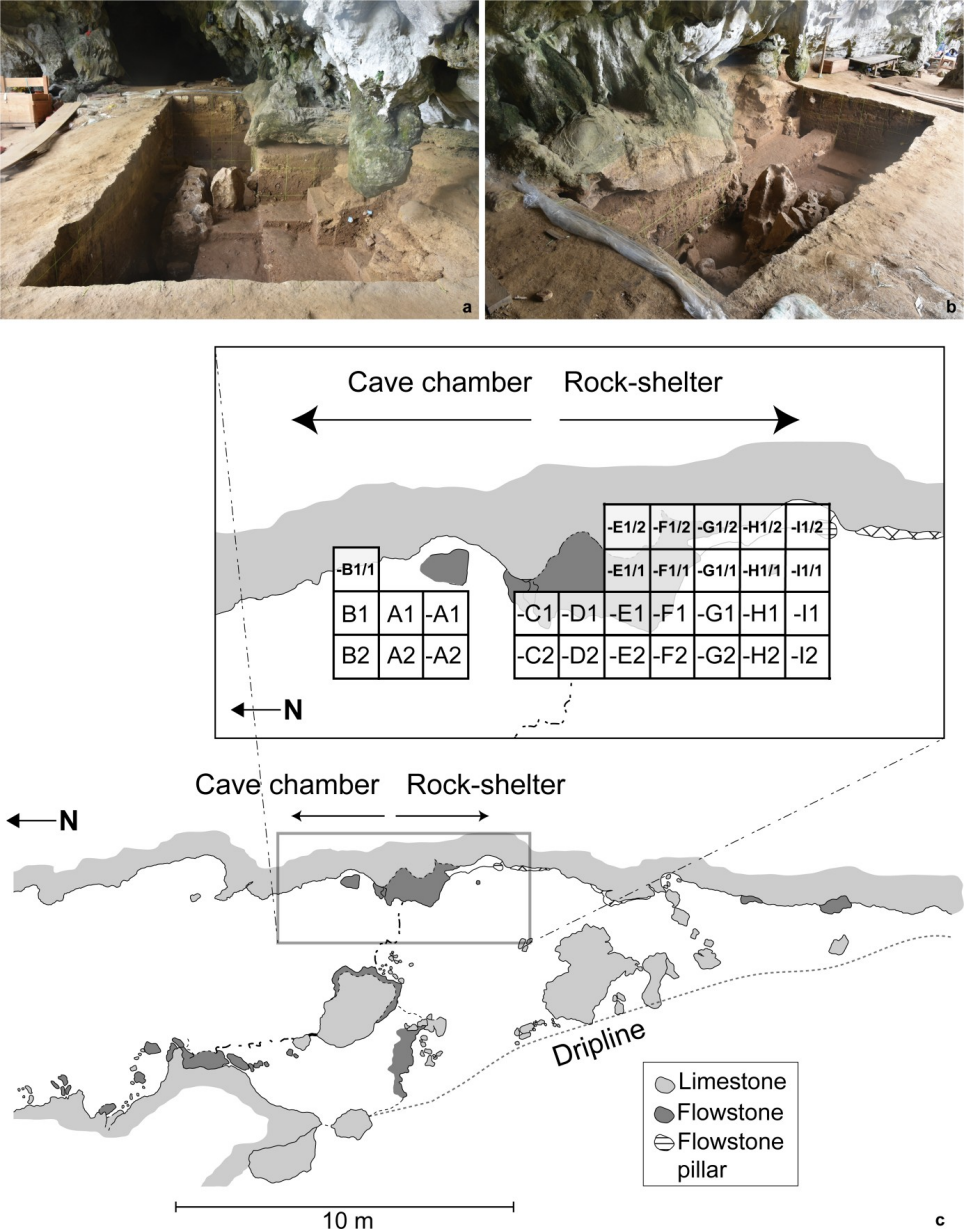
Excavations at Leang Bulu Bettue. (a-b) overview of the trench in the rock-shelter area (2017); (a) viewed from south to north; (b) viewed from northwest to southeast. (c) site plan showing the layout of the excavated squares in the rock-shelter and cave (2013–15, 2017–18).

Leang Bulu Bettue has been the focus of an annual program of joint Indonesian-Australian excavations carried out between 2013–15 and 2017–19. This work has uncovered a long sequence of stratified archaeological deposits inside the cave mouth and in the adjoining shelter (Fig 2). We excavated the deposit by stratigraphic layer using arbitrary 10 cm-deep spits, with *in situ* archaeological findings (e.g., stone artefacts, bones) measuring >10 mm in maximum dimension 3D-plotted using a total station. We wet-sieved cultural sediments on-site using 3 mm and 1 mm screens.

**Fig 3:**
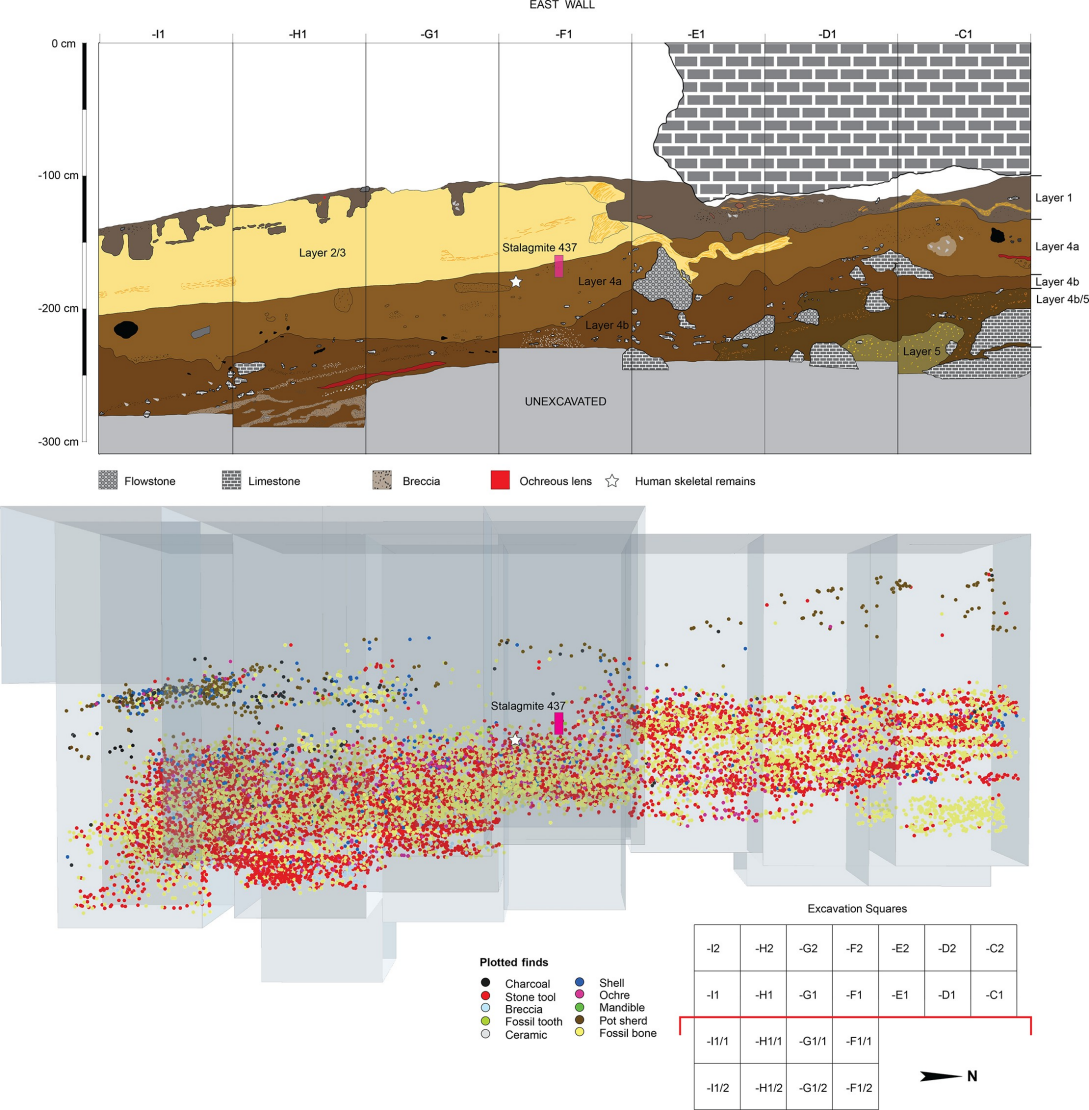
Stratigraphy and archaeological findings at Leang Bulu Bettue (2017). Top: East wall profile of the 2017 rock-shelter excavations, showing the stratigraphic sequence (note: Layers 4c-f are not visible here). Bottom: Spatial distribution of stone artefacts, faunal remains, and other findings recovered during the 2015–17 excavations, color-coded by stratigraphic layer (prepared using *ArcScene*). The location of the modern human right maxilla (Maros-LBB-1a) in relation to Stalagmite 437 is indicated by a white star.

A number of findings from these excavations are reported elsewhere [10; see also 32,33]. Here we briefly describe the stratigraphic sequence and cultural remains pertaining to the uppermost Late Pleistocene deposits (Layers 1–5), relevant to the present study. We have partitioned this undisturbed sequence of sedimentary layers (Fig 3) into two discrete human occupation phases: Phase II: Historical (<1790 A.D.) and ‘Neolithic’ (1.7–1.6 ka cal BP); and Phase I: MIS 3/2 (~50–16 ka). The Late Pleistocene cultural deposits of Phase I consist of silty clays with dense archaeological findings (Layers 4a-e). These sedimentary units are up to 1.5 m thick and span ~29.5–16 ka. Below this sequence is Layer 4f, a 50 cm-thick sandy clay layer dated to around 40–30 ka. Below Layer 4f is Layer 5, a 50 cm-thick sandy clay with relatively few cultural remains and faunal materials. Layer 5 has an estimated age of 50–40 ka.

The Late Pleistocene human skeletal remains described in this paper were excavated *in situ* during the 2017 field season. They were recovered from the upper part of the stratified and undisturbed Layer 4a (Fig 3), a ~70 cm thick moderate yellowish brown (10YR5/4) slightly sandy ‘mud’ (silt = 50.3%, clay = 32.2%). No other human remains have been found in Layer 4a. The skeletal elements were recovered in close association amidst profuse remains of what we consider to be ‘domestic’ activities, including lithic debris from stone artefact production and fragments of burnt animal bones reflecting food preparation and consumption. We encountered no evidence for a burial in Layer 4a. We also observed no clearly associated features or findings that could reasonably be interpreted as indicative of special contexts for the disposal of the human remains. The recovery of these isolated skeletal materials could be suggestive of the presence of burials in as-yet unexcavated portions of Layer 4a at the site.

The richest cultural and faunal assemblages excavated at Leang Bulu Bettue are found in Layer 4a [10]. The lithic technology used by the Layer 4a inhabitants was focused on chert reduction. It involved two stone-flaking techniques—direct freehand hard-hammer percussion; and anvil-supported bipolar percussion, where the blank was supported on a hard surface and the top edge struck, initiating flakes from the struck edge and the anvil support [10]. The dense faunal assemblage is dominated by shells of freshwater gastropods, mostly *Tylomelania perfecta*. The most frequently represented mammal remains are those of the bear cuscus (*Ailurops ursinus*), and various rodents. Sulawesi warty pig (*S*. *celebensis*) is the largest animal represented in Layer 4a. These ~40–85 kg wild suids are endemic to Sulawesi, although there is some evidence to suggest the species was translocated to various other Wallacean islands (and possibly further afield) in late prehistory [34]. We also uncovered findings indicative of symbolic behaviour in Layer 4a, including a drilled pendant made from an *A*. *ursinus* phalanx and several engraved stone artefacts, some of which consist of flaked chert artefacts with geometric motifs incised into cortical surfaces [10]. Two stone ‘plaquettes’ engraved with what seem to be figurative motifs have also been recovered [33]. Evidence for pigment use in Layer 4a includes utilised mineral colorant nodules and ochre residues on the surfaces of both stone and bone tools [10].

### Antiquity of the human remains

Prior dating work [10,32], and new evidence presented here, allows us to constrain the age of Layer 4a to between 24.8 and 16 ka, thus broadly within the timespan of the LGM. As far as we have been able to ascertain no charcoal is preserved in the Late Pleistocene deposits at Leang Bulu Bettue. Hence, the chronology for Layer 4 ais based on four independent dating methods: 1) U-series isotope analysis undertaken on vertical, still-emplaced stalagmites exposed during excavations of Layer 4a/b; 2) AMS ^14^C-dating of *T*. *perfecta* shells recovered *in situ* from Layer 4a; 3) laser ablation U-series dating of a pig tooth excavated from Layer 4a; and 4) optical dating (post-infrared infrared stimulated luminescence [pIRIR]) of feldspars from Layer 4a.

#### Stalagmite chronology

**Fig 4:**
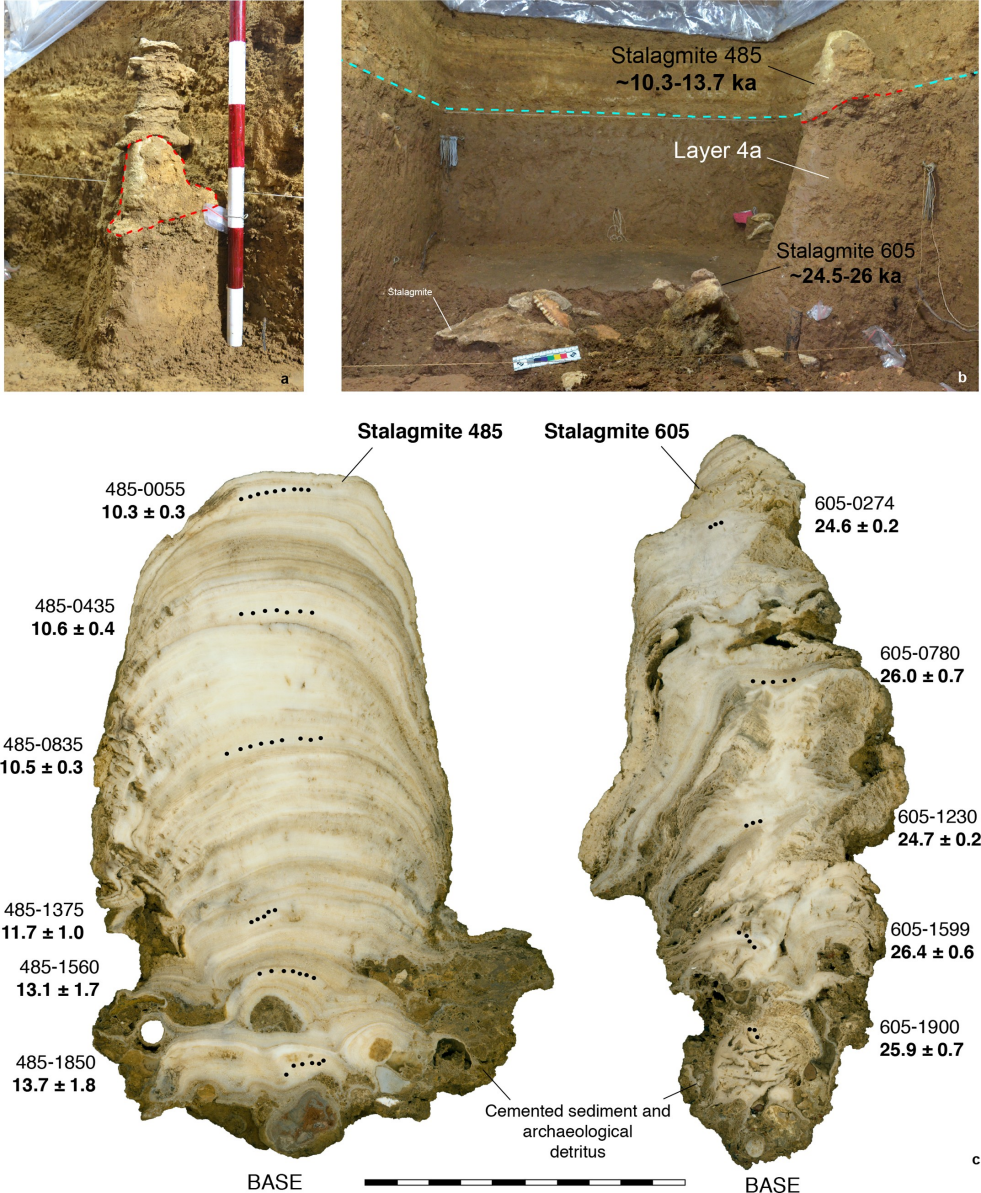
Stalagmites dated using U-series analysis at Leang Bulu Bettue. (a) Stalagmite 485 (scale is in 10 cm increments). The *in situ* speleothem comprises distinct lower and upper sections. We only dated the lower section (highlighted by a broken red line). This part of the stalagmite has a diameter of 13.5 cm. It grew to a height of 18 cm on a 10 cm-thick pedestal of cemented sediment and archaeological detritus, which includes shell, bone, ochre fragments and stone artefacts. During excavation the lower section of the stalagmite was left *in situ* on a plinth of Layer 4a sediments; (b) Stalagmites 485 (lower section) and 605 *in situ*. Stalagmite 485, the base of which is highlighted by a broken red line, grew atop Layer 4a. The broken blue line shows the boundary between Layer 4a and overlying Layer 3f. Stalagmite 485 grew between 13.7 to 10.3 ka, providing a minimum age for Layer 4a. Stalagmite 605 formed on top of Layer 4b between 26 to 24.5 ka; (c) cross-sections of Stalagmites 485 and 605 showing U-series sub-sample locations and dating results (scale is in 10 mm increments).

**Fig 5:**
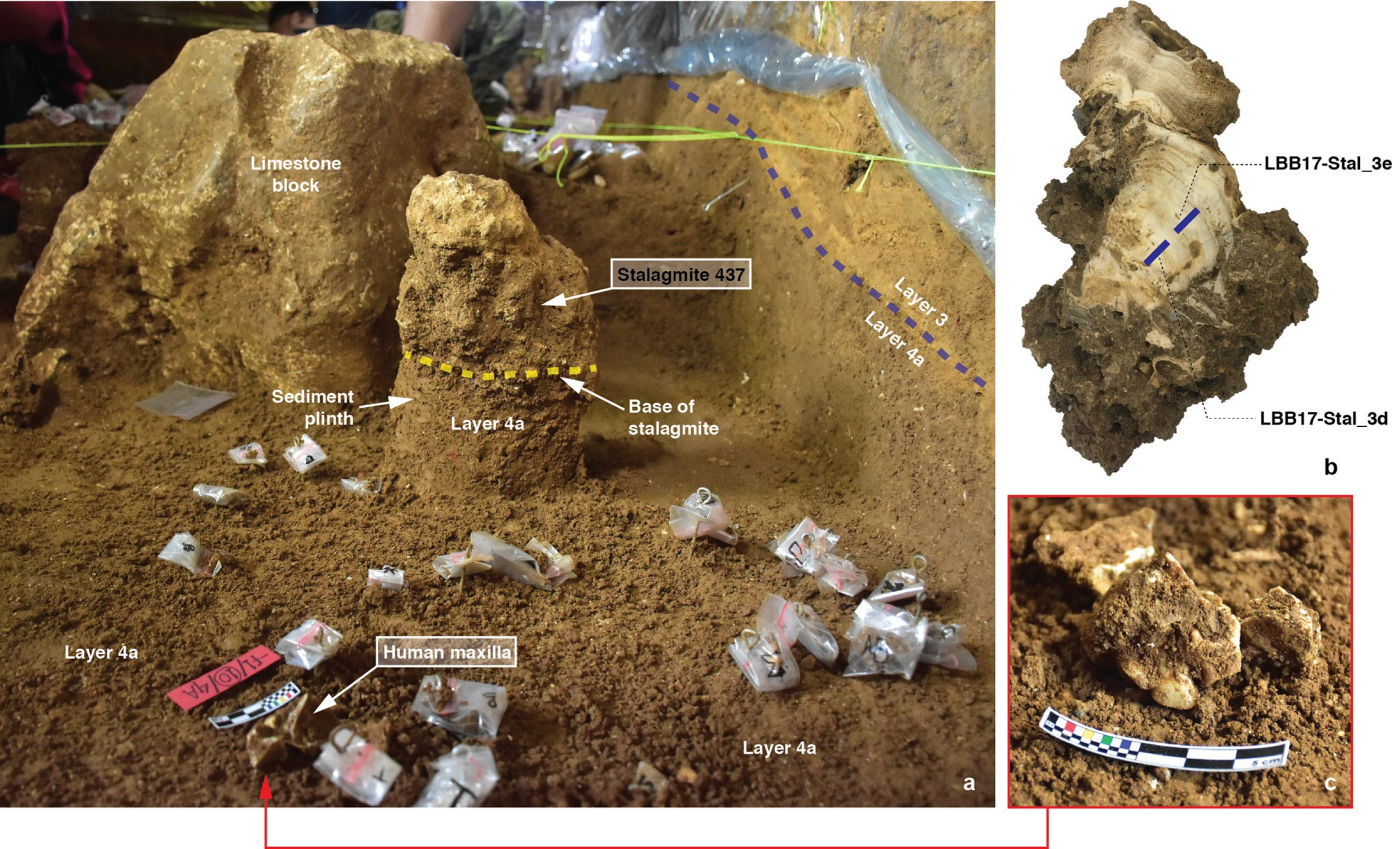
Stalagmite dated using U-series analysis at Leang Bulu Bettue. (a) Stalagmite 437 *in situ* within the trench under excavation–during the excavation the stalagmite was left *in situ* on a plinth of unexcavated Layer 4a sediments. The blue dashed line shows the boundary between Layer 4a and overlying Layer 3; (b) cross-section of Stalagmite 437 showing the locations of U-series sub-samples LBB17-Stal_3d and LBB17-Stal_3e, which were used to determine the growth age of the speleothem; (c) human maxilla fragments (Maros-LBB-1a) *in situ* in Layer 4a below the base of Stalagmite 437.

We dated three speleothem samples associated with Layer 4a: Stalagmite 485 (Fig 4) and Stalagmite 437 (Fig 5), which both formed above this layer, and Stalagmite 605, which formed below it (Fig 4). The results of U-series dating of these three stalagmites are provided in Table 1. Following collection in the field, the stalagmites were sawn longitudinally. In the case of Stalagmites 485 and 605, trace element analyses were conducted along the growth axes to identify calcite layers with the greatest U-series dating potential. Trace element analysis was not undertaken on Stalagmite 437. U-series isotope analyses of the three stalagmites were carried out in the Radiogenic Isotope Facility of the University of Queensland, Brisbane, on a Nu Plasma multi-collector inductively coupled mass spectrometer (MC-ICP-MS), following chemical separation procedures and MC-ICP-MS analytical protocols described elsewhere [35,36]. The ^230^Th/^234^U ages were calculated using Isoplot EX 3.75 [37] and half-lives of 75,690 years (^230^Th) and 245,250 years (^234^U) [38].

**Table 1:** U-series dating results for stalagmites from Leang Bulu Bettue.

Stalagmite No	Sub-Sample	Sub-sampling depth (mm)	Wt (g)	^238^U (ppm)	^232^Th (ppb)	(^230^Th/^232^Th)	±2σ	(^230^Th/^238^U)	±2σ	(^234^U/^238^U)	±2σ	Uncorrected Age (ka)	±2σ	Corrected Age (ka)	±2σ	Initial (^234^U/^238^U)	±2σ
485	0055	5.5	0.04533	0.947	7.70	18.16	0.11	0.0486	0.0003	0.5242	0.0004	10.77	0.07	**10.29**	**0.26**	0.5091	0.0008
485	0435	43.5	0.03640	0.786	9.30	13.17	0.10	0.0513	0.0004	0.5278	0.0007	11.32	0.09	**10.64**	**0.37**	0.5119	0.0012
485	0835	83.5	0.04809	0.785	8.24	14.50	0.15	0.0502	0.0005	0.5247	0.0006	11.14	0.12	**10.52**	**0.34**	0.5089	0.0011
485	1375	137.5	0.02775	0.753	22.6	6.11	0.08	0.0605	0.0008	0.5287	0.0008	13.480	0.19	**11.73**	**0.95**	0.5088	0.0026
485	1560	156	0.02380	0.94	50.27	4.12	0.04	0.0722	0.0007	0.5311	0.0006	16.28	0.17	**13.1**	**1.7**	0.5062	0.0045
485	1850	185	0.02406	0.920	50.64	4.11	0.06	0.0744	0.0011	0.5265	0.0009	17.01	0.29	**13.7**	**1.8**	0.5003	0.0048
437	e	76	0.00218	0.562	55.82	3.44	0.07	0.1126	0.0023	0.6180	0.0013	22.44	0.52	**17.4**	**2.8**	0.5832	0.0086
437	d	96	0.00363	0.622	44.94	4.66	0.06	0.1110	0.0015	0.6300	0.0013	21.58	0.32	**18.0**	**2.0**	0.5998	0.0060
437	c	123	0.00596	0.576	145.52	2.74	0.03	0.2284	0.0029	0.6686	0.0014	47.54	0.79	34.9	7.7	0.5953	0.0231
437	b	127	0.00581	0.576	274.07	2.37	0.02	0.3724	0.0032	0.6660	0.0016	99.6	1.7	72	22	0.5049	0.0696
437	a	129	0.00479	0.643	459.04	2.35	0.02	0.5529	0.0035	0.6795	0.0020	unable to calculate	-	277	924	0.2394	0.6739
605	0274	27.4	0.01997	1.334	3.78	111.42	0.77	0.1039	0.0007	0.5284	0.0010	24.73	0.20	**24.56**	**0.23**	0.4942	0.0012
605	0780	78	0.01840	1.82	37.85	16.50	0.08	0.1128	0.0005	0.5272	0.0008	27.30	0.16	**26.04**	**0.74**	0.4883	0.0021
605	1230	123	0.01792	1.47	4.53	102.07	0.57	0.1038	0.0006	0.5257	0.0008	24.86	0.16	**24.68**	**0.19**	0.4911	0.0009
605	1599	159.9	0.01928	1.064	16.41	22.43	0.20	0.1141	0.0010	0.5321	0.0008	27.34	0.28	**26.41**	**0.60**	0.4937	0.0017
605	1900	190	0.00775	1.340	26.01	17.86	0.11	0.1142	0.0007	0.5380	0.0012	27.01	0.21	**25.86**	**0.69**	0.5004	0.0022

This table contains the results of U-series disequilibrium dating of three stalagmites: Stalagmites 485, 437, and 605. Note: Ratios are activity ratios calculated from the atomic ratios. Errors are at 2δ level. The ages are calculated using Isoplot 3.75 Program [37] with decay constants from Cheng *et al*. [38]. Corrected ages were calculated assuming initial/detrital ^230^Th/^232^Th activity ratio equal 0.825 (± 50%) (the bulk-Earth value, which is the most commonly used for initial/detrital ^230^Th corrections). Sub-sampling depth denotes the depth at which each sample was collected from the tip of the stalagmite along the main growth axis.

Stalagmite 485 (height: 35.6 cm, basal width: 15.7 cm) and Stalagmite 605 (height: 18 cm, diameter: ~11 cm) were exposed during the 2013 excavations. Initial U-series dating results for these speleothems are reported elsewhere [10] (Fig 4). The two stalagmites were uncovered during excavation of Square A1 inside the cave mouth ~500 cm north of the human skeletal remains find spot. Both samples comprise *in situ* upright stalagmites. Stalagmite 485 is the stratigraphically youngest sample. This speleothem formed on the upper surface of Layer 4a. Stalagmite 605 grew on the upper surface of underlying Layer 4b. It was buried at a later stage by the accumulation of Layer 4a, suggesting there was a depositional hiatus between these layers. Both stalagmites formed on top of ~10–15 cm-thick pedestals of cemented sediment and archaeological detritus. Archaeological inclusions in the pedestals consist of shell, bone, ochre, and lithic artefacts. These pedestals appear to have formed as a result of calcium carbonate-enriched water dripping from the overhead ceiling and forming a hardened mass on the soft sedimentary deposits of the cave floor.

Stalagmite 437 is a vertical and still *in situ* stalagmite that formed atop the sloping upper surface of Layer 4a in the rock-shelter area (Fig 5). Stalagmite 437 is irregular in form. The speleothem portion is 8 cm in height and 10 cm in width, and 9.5 cm thick at the base. Below the basal growth layers was a pedestal of cemented archaeological deposit measuring 17 cm in depth by 15 cm in width by 14 cm in thickness and containing characteristic Layer 4a findings, such as *T*. *perfecta* shells and flaked chert artefacts. The human skeletal remains were recovered a distance of 38 cm to the south of Stalagmite 437 and 3.5 cm below the base of this intact speleothem.

As has been previously noted, Stalagmite 485 is located immediately above Layer 4a and thus the basal growth age of this speleothem provides us with a minimum age for this archaeological horizon. Six (n = 6) U-series ages estimates (sub-samples 0055, 0435, 0835, 1375, 1560, 1850) were calculated along the main growth axis of the speleothem (Fig 4, Table 1). Their ^230^Th/^232^Th activity ratios range between 4.11 and 18.16. The cleanest samples (n = 3) are located towards the tip of the speleothem, with analytically indistinguishable ages of 10.3 ± 0.3 to 10.5 ± 0.4 ka. The other samples (n = 3) are located towards the base of the speleothem and their ages range between 11.7 ± 1.0 to 13.7 ± 1.8 ka. The oldest minimum age for Layer 4a is determined by sub-sample 1850, with a detrital-^230^Th corrected age of 13.7 ± 1.8 ka. Layer 4a is therefore at least 11.9 ka and could be at least as old as 15.5 ka. This also corresponds to the base of the speleothem having formed immediately above Layer 4a.

Stalagmite 437 (UQ lab code: Sample LBB17-Stal_3) also lies immediately above Layer 4a. Five U-series age estimates (LBB17-Stal_3a-e) were calculated along the growth axis of the lower part of the speleothem (Table 1). All have relatively low ^230^Th/^232^Th activity ratios ranging between 2.35 and 4.66, significantly lower than is the case in Stalagmite 485. It is therefore evident from this observation that these sub-samples are heavily contaminated with detrital components. Sub-samples LBB17-Stal_3a-c have been rejected on the basis of their low ^230^Th/^232^Th activity ratios of between 2.35 and 2.74. The corrected ages for these samples are also older than the maximum age for Layer 4a (see below). Sub-samples LBB17-Stal_3d and LBB17-Stal_3e (Fig 5) have slightly higher ^230^Th/^232^Th activity ratios of 4.66 and 3.44. This is consistent with the age sequence defined by sub-samples of Stalagmite 485, which are overall purer. The oldest minimum age for Layer 4a is now provided by sub-sample LBB17-Stal_3d with a detrital-^230^Th corrected age of 18 ± 2 ka. Layer 4a is therefore at least 16 ka, but it could be at least as old as 20 ka. This finding is also consistent with the base of the speleothem having formed immediately above Layer 4a.

Stalagmite 605 is located below Layer 4a and has formed immediately above Layer 4b. Five (n = 5) U-series age estimates (0274, 0780, 1230, 1599, 1900) were calculated along the growth axis of the speleothem (Fig 4, Table 1). The sub-samples are all relatively clean, with ^230^Th/^232^Th activity ratios ranging between 16.50 and 111.42. Their ages range between 24.6 ± 0.2 ka to 26.4 ± 0.6 ka. The youngest maximum aged for Layer 4a is determined by sub-sample 0274 with a detrital-^230^Th corrected age of 24.6 ± 0.2 ka, suggesting the layer could be younger than this age. This also corresponds to the tip of the speleothem having formed before the deposition of Layer 4a. Thus, based on the stratigraphical context of the above three stalagmites, we infer that Layer 4a can be securely bracketed into 18 ± 2 to 24.6 ± 0.2 ka.

#### Radiocarbon dating

We conducted AMS ^14^C dating on a *T*. *perfecta* shell that was excavated *in situ* from near to the top of Layer 4a inside the cave (131 cm depth, Squares A1 and A2). This shell was located ~10 cm below the pedestal of cemented archaeological detritus underlying Stalagmite 485. It yielded an AMS ^14^C age (Wk-37742) of 18,126 ± 51 BP or 22.2–21.9 cal ka BP at 2σ. This date was calibrated in OxCal 4.4 using an unconstrained mix of the IntCal20 and SHCal20 revised calibration curves, as recommended in Hogg et al. 2020 [39]. With regards to this age estimate on *T*. *perfecta* shell, it has not been possible thus far to calculate the magnitude of the freshwater reservoir (or hardwater effect) in Maros-Pangkep [27,31].

#### Optical dating

As reported elsewhere [32; see also 10], we conducted pIRIR-dating on Layer 4a feldspars (LBB-I) collected from the south wall of Square A2 at about 8 cm below the level of sample Wk-37742, yielding a depositional age of 21.4 ± 2.5 ka (68.2% probability [1σ]) for Layer 4a. Reported at the 95.4% confidence interval, error ranges for the pIRIR feldspar (16.4–26.4 ka) age suggest the upper part of Layer 4a is at least 16 ka, which, again, is consistent with the U-series ages obtained from the two *in situ* stalagmites that developed immediately on top of this layer (Stalagmites 485 and 437).

#### U-series dating of fossil tooth

We conducted laser ablation U-series dating on a suid molar recovered from Layer 4a (LBB3 1B-14B). The specimen was collected at a depth of 156 cm. Results indicate a minimum age of 15.9 ± 0.5 ka (1σ) for this tooth. This is also consistent with the stalagmite chronology, which indicates that Layer 4a dates to more than 16 ka.

#### Summary of dating results for Layer 4a

Previously, we inferred an age of 26–22 ka for Layer 4a based on four independent dating methods [10]. In that prior work, the ^14^C date for the freshwater shell (Wk-37742) from below Stalagmite 485 was used as a reasonable estimate for the upper age of Layer 4a (~22.3 cal ka BP). Based on the uncertainties introduced owing to the freshwater reservoir effect, however, we now prefer to rely on the U-series stalagmite chronology, including new dates reported for the first time here. This suggests an age of 24.8–16 ka for Layer 4a in which the human remains were recovered.

### Summary of the human remains

**Fig 6:**
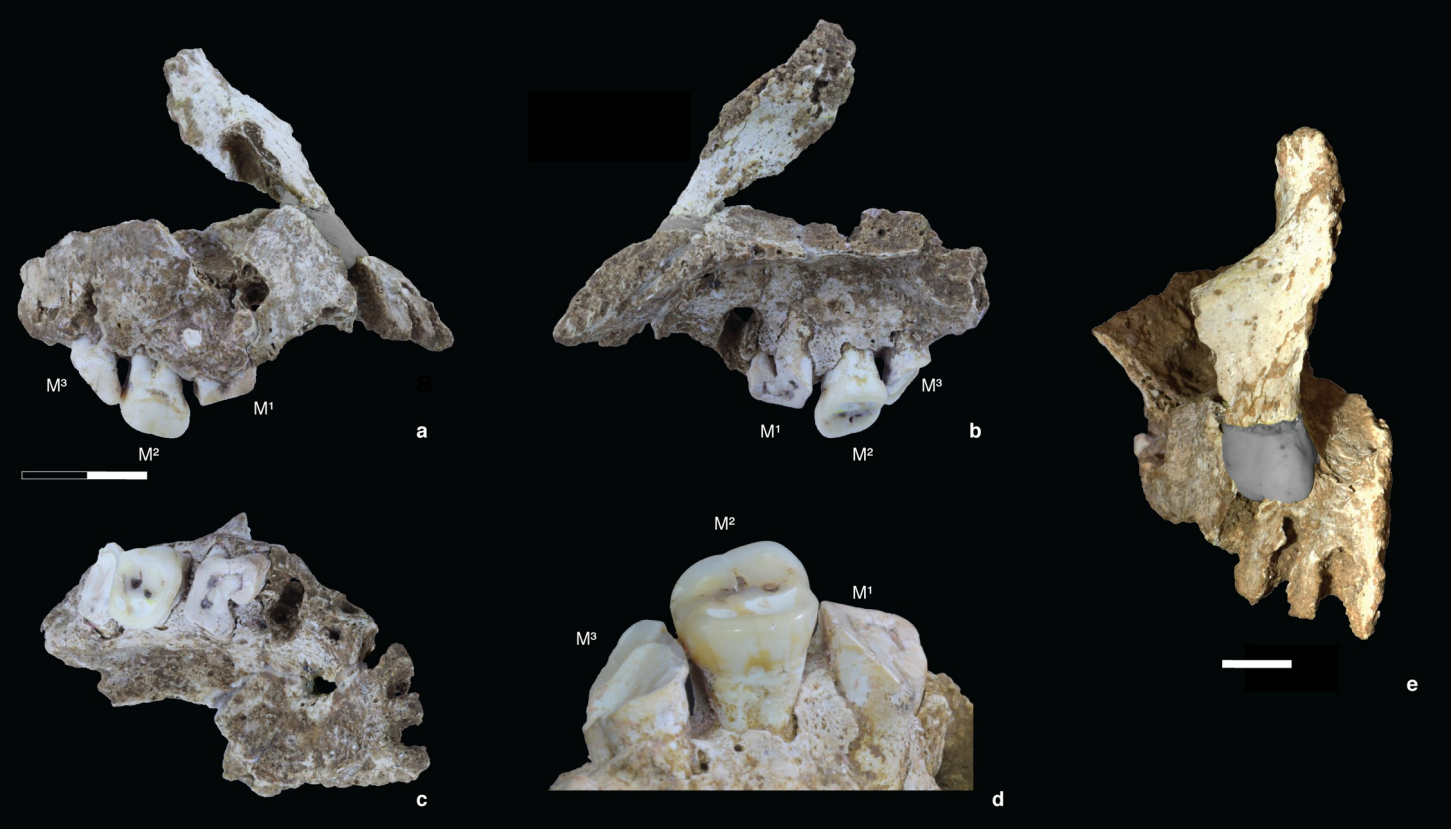
Right maxilla and frontal process (Maros-LBB-1a) from Layer 4a at Leang Bulu Bettue. (a-b) right and left lateral views of the right maxilla (after reconstruction). The small point of contact between the dental portion and nasal pillar is obscured by glue and plasticine; (c) inferior view of the right palate; (d) detail of the lingual sides of the extant first to third right upper molars (M^1^ to M^3^), showing the extreme degree of occlusal attrition on the M^1^ and M^3^. The M^1^ had lost its crown by extreme wear and there are abscess cavities around the root tips, owing to the exposure of dental pulp cavities. The M^3^ retains its enamel only at the mesiobuccal corner. Only the M^2^ has a normal occlusal plane. The M^2^ still retains much of its occlusal enamel but is considerably over-erupted, suggesting that its opposing tooth (M_2_) had been lost while the individual was alive; (e) anterior view of the reconstruction of the right maxilla and frontal process. The scale in (a-c) is in 10 mm increments; in (e) the scale bar is 10 mm. Photo credits: Ratno Sardi (a-d); David Bulbeck (e).

The key specimen, designated Maros-LBB-1a, consists of a nearly complete palate and frontal process of the right maxilla (Figs 6 and S1). The former consists of several fragments (combined weight 18 grams) excavated *in situ* in Square -F1 (Spit 10), and the latter was recovered from wet-sieving of the same square and spit. An evident point of contact between the elements was discerned at the inferior nasal aperture, leading to the inference of a vertically short nasal aperture, and a reconstruction with plasticine inserted to simulate the missing maxillary bone.

The right maxilla contains the first to third right upper molars (M^1^, M^2^, and M^3^). In addition, a left maxilla fragment weighing 1 gram was recovered from nearby Square E1 (Spit 11), and two conjoining mandible fragments (combined weight 3 grams) were also excavated from Square E1 (Spit 12) and Square G2 (Spit 11); however, these specimens are too fragmentary for analysis and hence they are not considered further here. All of these skeletal materials (including the undiagnostic elements) are considered to come from a single individual. The Maros-LBB-1a skeletal remains (and associated fragments) are unburnt and their partly mineralised condition is in keeping with the majority of Late Pleistocene faunal elements excavated so far from Layer 4a.

## Methods

The Indonesian field research was authorised by Indonesia’s State Ministry of Research and Technology (RISTEK) and was conducted in collaboration with counterpart institution Pusat Penelitian Arkeologi Nasional (ARKENAS), Jakarta, Indonesia. All necessary permits were obtained from Indonesia’s State Ministry of Research and Technology for the described study (Permit No: 154/SIP/FRP/E5/Dit.KI/VII/2017), which complied with all relevant regulations. The human skeletal remains from Leang Bulu Bettue (specimen number: Maros-LBB-1a) are permanently stored at the premises of the Balai Arkeologi Sulawesi Selatan (Makassar Archaeology Office in South Sulawesi) in Makassar, South Sulawesi. Requests to access collections for study, including databases and catalogs of finds, should be directed in the first instance to the directors of Pusat Penelitian Arkeologi Nasional (ARKENAS) (http://arkenas.kemdikbud.go.id/#1) and Balai Arkeologi Sulawesi Selatan (https://balar-sulsel.kemdikbud.go.id).

### Morphological analysis

The methodology for the analysis of Maros-LBB-1a closely followed the techniques applied by DB to Gua Cha in Peninsular Malaysia [40] so as to assist comparison with this substantial assemblage of mid-Holocene burials from an Island Southeast Asian rainforest environment. The three *in situ* teeth were measured for their maximum mesio-distal and bucco-lingual diameters and also these diameters at the cemento-enamel junction. Cranial measurements followed the definitions in Bräuer [41] and Howells [42]. Measurements of Maros-LBB-1a were taken with a Kincrome electronic calliper accurate to 0.01 mm (generally rounded off to the closest tenth of a millimetre). Oral pathology was recorded following Patterson [43], although indications of periodontal disease are inferred here following Tayles [44:238]. Dental morphology features recorded were those of the Arizona State University (ASU) system [45] including reference to standard plaques illustrated in that work and Hillson [46] for photographs of Carabelli’s cusp development. Cranial morphology was recorded following Larnach and Macintosh [47].

### Taxonomy

**Fig 7:**
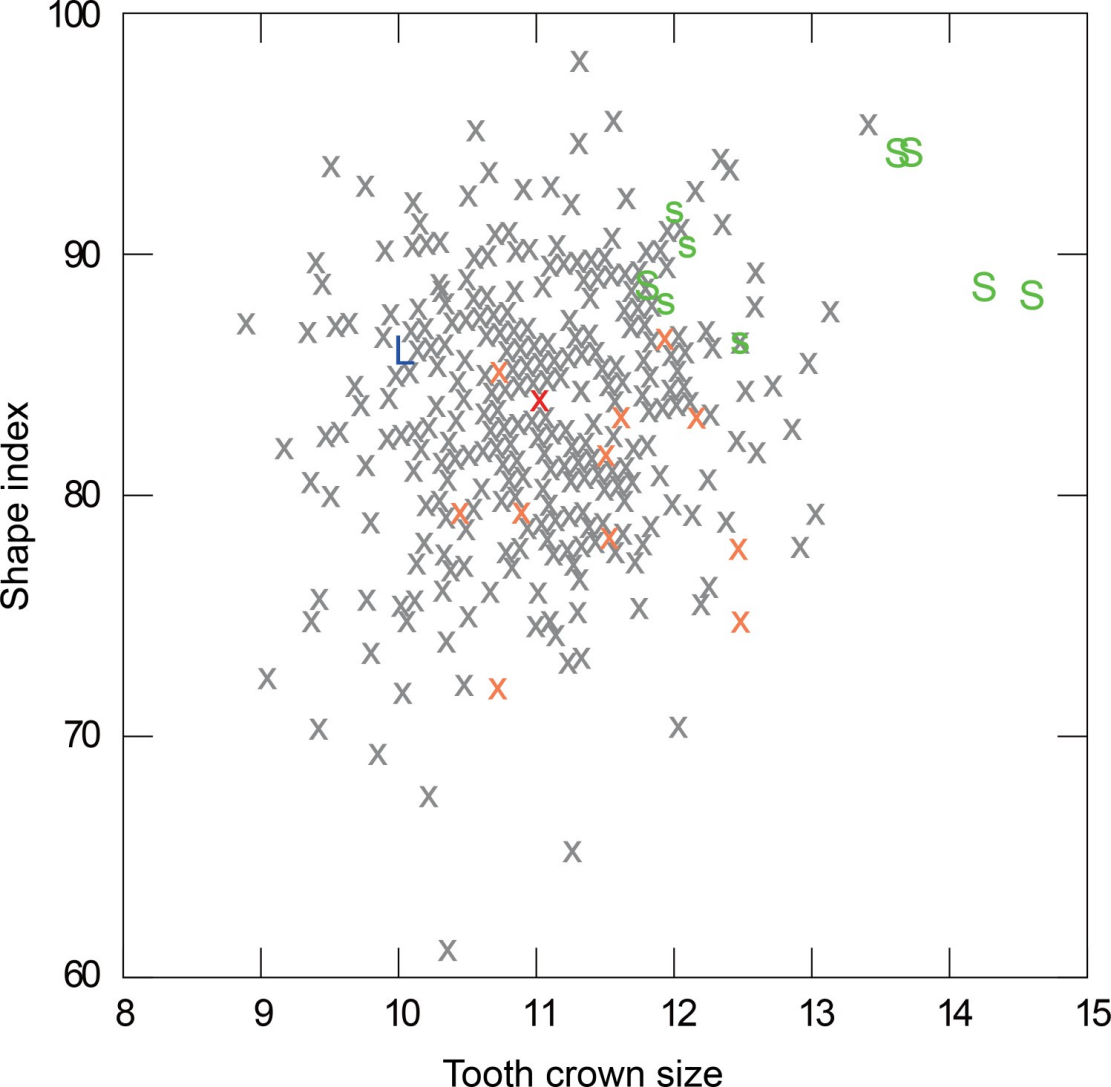
M^2^ crown size and shape in Maros-LBB-1a, other *H*. *sapiens*, Javanese *H*. *erectus*, and *H*. *floresiensis*. Crown size = SQRT (BL diam. x MD diam.); crown shape index = (MD diam./BL diam.) x 100. Red cross: Maros-LBB-1a (measurement by Y. Kaifu); orange crosses: Prehistoric Indonesian *H*. *sapiens* (Java and Flores) (N = 11); grey crosses: Global *H*. *sapiens* (Asia, Australia/Melanesia, Africa, Europe) (N = 363); green “S” and “s”: Sangiran *H*. *erectus* (S, older subgroup; s, younger subgroup) (N = 9); blue “L”: *H*. *floresiensis* (LB1). Y. Kaifu carried out all measurements. For data sources for comparative *H*. *sapiens* sample, see Table 2.

The Leang Bulu Bettue individual (Maros-LBB-1a) is clearly different from two representative pre-modern hominin groups in the region, *Homo erectus* from Java and *H*. *floresiensis* from Flores. The latter exhibits a distinctly protruded maxillary process of the malar, as reflected by an anteriorly positioned lateral nasal margin and laterally faced bone surface beside it, which is marked posteriorly by the formation of an infraorbital (maxillary) sulcus, modest alveolar prognathism, a mesiodistally elongated M^2^ crown, and a lingual molar root that diverges strongly medially [48–50]. Maros-LBB-1a displays enhanced alveolar prognathism and the relatively short M^2^ crown is outside the range of variation for *H*. *erectus* (Fig 7). It is also divergent from *H*. *floresiensis* (Fig 7). Theoretically, Maros-LBB-1a could belong to a member of the now-extinct and apparently geographically widely dispersed hominin Denisovan branch, but no Denisovan cranial remains have been identified [51] to allow this possibility to be tested. Rather, as described below, Maros-LBB-1a clearly falls within the morphological range of *H*. *sapiens* in the region and so is assigned to AMH.

**Table 2:** Comparative *Homo sapiens* sample (see Fig 7).

	Remarks	N^a^	Repository^b^
**Prehistoric Southeast Asia**			
Flores*	Aimere, Gua Alo, Gua Nempong, Liang Bua, Liang Momer, Liang Toge, Liang X	9	NBC, ARKENAS
Java*	Hoekgrot, Wajak	3	NBC
Malaysia*	Guar Kepah	19	NBC
Vietnam*	Mai Da Dieu, Mai Da Nuoc, Hang Chim, Dong Cang, Con Co Ngua	73	IAH
**Australia/Melanesia**			
New Guinea*		30	AMNH, MH
Australia/Tasmania Aboriginal Australian*		19	AMNH
**Southeast Asia**			
Philippine ‘Negrito’*		20	MH
Others	Andaman, Indonesia, Malaysia, Nicobar, Philippine, Singapore, Thailand	57	AMNH, MH
**Northeast Asia**			
Northeast Asia	China, Chukuci, Korea, Mongol, Yukagir	18	AMNH
**Africa**			
‘Bushman’		17	AMNH, MH
African ‘Pygmy’*		20	MH
South Africa	Excluding ‘Bushman’	26	AMNH
East Africa		45	AMNH
West Africa	Excluding ‘Pygmy’	55	AMNH
**Indo/Europe**			
India		6	AMNH
German		65	AMNH
Others	Hungary, Poland, Sweden	8	AMNH
Total		490	

*Samples included in the EFAs.

^a^Number of individuals.

^b^NBC = Naturalis Biodiversity Center, Leiden; ARKENAS = National Research and Development Centre for Archaeology, Jakarta; AMNH = American Museum of Natural History, NY; MH = Musee de l’Homme, Paris, IAH = Institute of Archaeology, Hanoi.

### Age and sex of Maros-LBB-1a

This AMH individual is evidently adult, as shown by the worn status of the extant M^3^ (Fig 6, Table 3). As reconstructed, the extant remains suggest an individual of small to medium size:

- estimated (right) nasal height of ~42 mm, similar to the averages recorded for Andaman Islander females and Khoisan males and females, but smaller than the averages recorded by Howells [42] for any other male or female series;
- estimated nasal breadth (based on doubling the extant right breadth) of 27 mm, which is similar to the averages recorded for Southwest Pacific females, and Polynesian/Micronesian, Ainu and Khoisan males and females [42].

**Table 3:** Right maxillary molar wear (Smith’s system, in [4,6]) and calculus development (after [43]) evident in Maros-LBB-1a.

Tooth	Occlusal wear	Calculus location	Calculus amount
First molar	4*	Radicular: disto-bucally and disto-lingually	Slight
Second molar	3	Coronal: mesio-buccally	Slight
Third molar	7	Radicular: entire buccal and distal surfaces, and disto-lingually	Moderate

*The mesio-lingual corner of the tooth was chipped off during life, making assessment of the tooth’s occlusal wear problematic.

**Fig 8:**
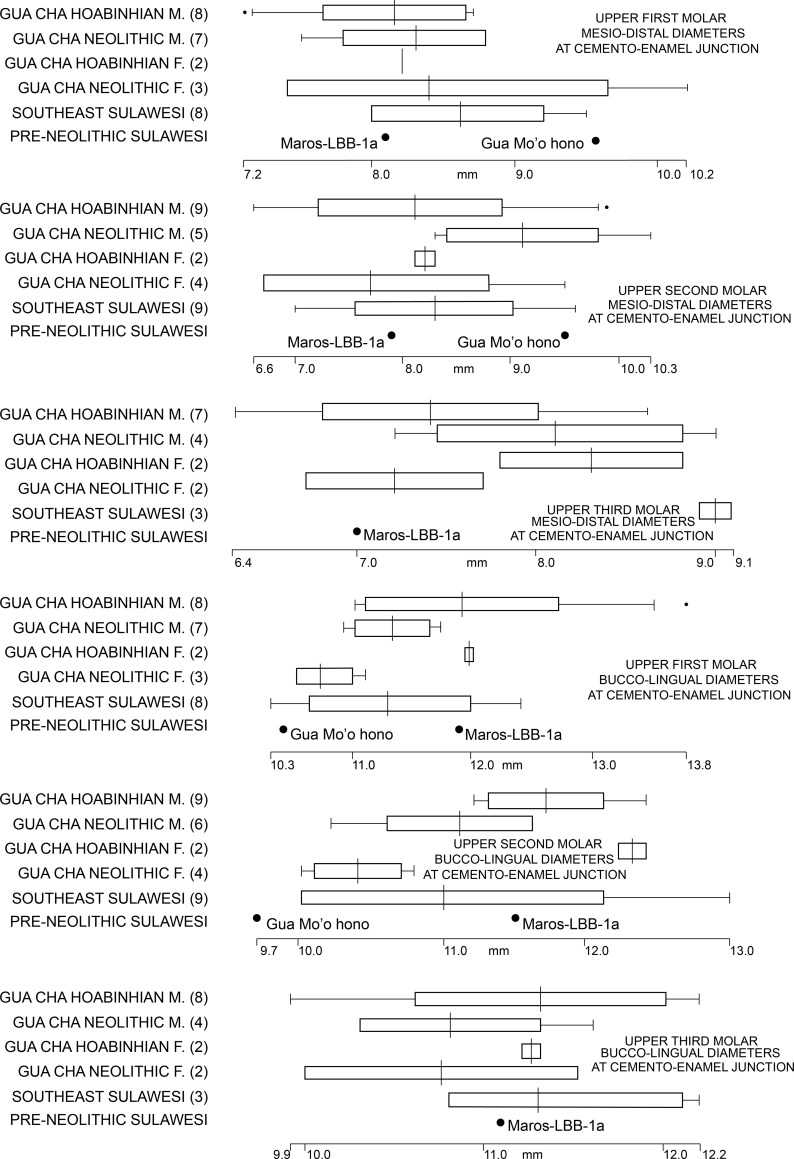
Comparative data for Island Southeast Asian upper molar diameters at the cemento-enamel junction. Sources: [40,53]; Table 4 (this paper).

The palate is of *medium* size [52] with its length and estimated breadth of respectively 60 and 64 mm producing a module of 38.4. The teeth are quite small in size, with available measurements that either fall reasonably close to the averages for 2^nd^ millennium CE burials from Southeast Sulawesi or below their recorded range (Table 4). However, the particularly small Maros-LBB-1a tooth diameters reflect loss of the bulkiest part of the crown due to advanced occlusal attrition (Fig 6, Table 3) and so we focus on the tooth diameters at the cemento-enamel junction. Doing this, and including comparative data from the (sexually and chronologically, comprehensively overlapping) Gua Cha teeth in Peninsular Malaysia, we see that the Maros-LBB-1a tooth diameters fall within the lower median of the comparative range (Fig 8).

**Table 4:** Diameters (mm) of the Maros-LBB-1a molars, with Southeast Sulawesi comparisons (from [54]; Bulbeck laboratory observations).

Tooth	Maros-LBB-1a		Gua Lampetia/Gua Andomo/Gua Sambangoala
	Measurement		Average (range)
First upper molar	Mesio-distal diameter	>>8.5*	10.4 (9.6–11.4)
Second upper molar	ˈ ˈ	9.8	9.7 (9.0–10.7)
Third upper molar	ˈ ˈ	9.2^#^	9.8 (9.5–10.0)
First upper molar	Bucco-lingual diameter	12.2	11.7 (10.6–12.6)
Second upper molar	ˈ ˈ	11.6	11.6 (10.9–13.4)
Third upper molar	ˈ ˈ	10.7*	11.5 (11.0–12.4)
First upper molar	Mesio-distal diameter at cemento-enamel junction	8.1	8.6 (8.0–9.5)
Second upper molar	ˈ ˈ	7.9	8.3 (7.0–9.6)
Third upper molar	ˈ ˈ	7.0	9.0 (8.9–9.1)
First upper molar	Bucco-lingual diameter at cemento-enamel junction	11.9	11.3 (10.3–12.4)
Second upper molar	ˈ ˈ	11.5	11.0 (10.0–13.0)
Third upper molar	ˈ ˈ	11.1	11.3 (10.8–12.2)

*Cannot be measured with any reliability due to advanced interproximal wear.

^#^Somewhat reduced from advanced occlusal wear.

In summary, sex is difficult to estimate for such fragmentary remains, especially without knowing the specimen’s comparative population. It is noteworthy, however, that the Tron Bon Lei individuals are surprisingly small in size, being ‘unique even by Pleistocene standards in the combination of small and narrow morphologies’ [2: p12].

### Oral disease

**Fig 9:**
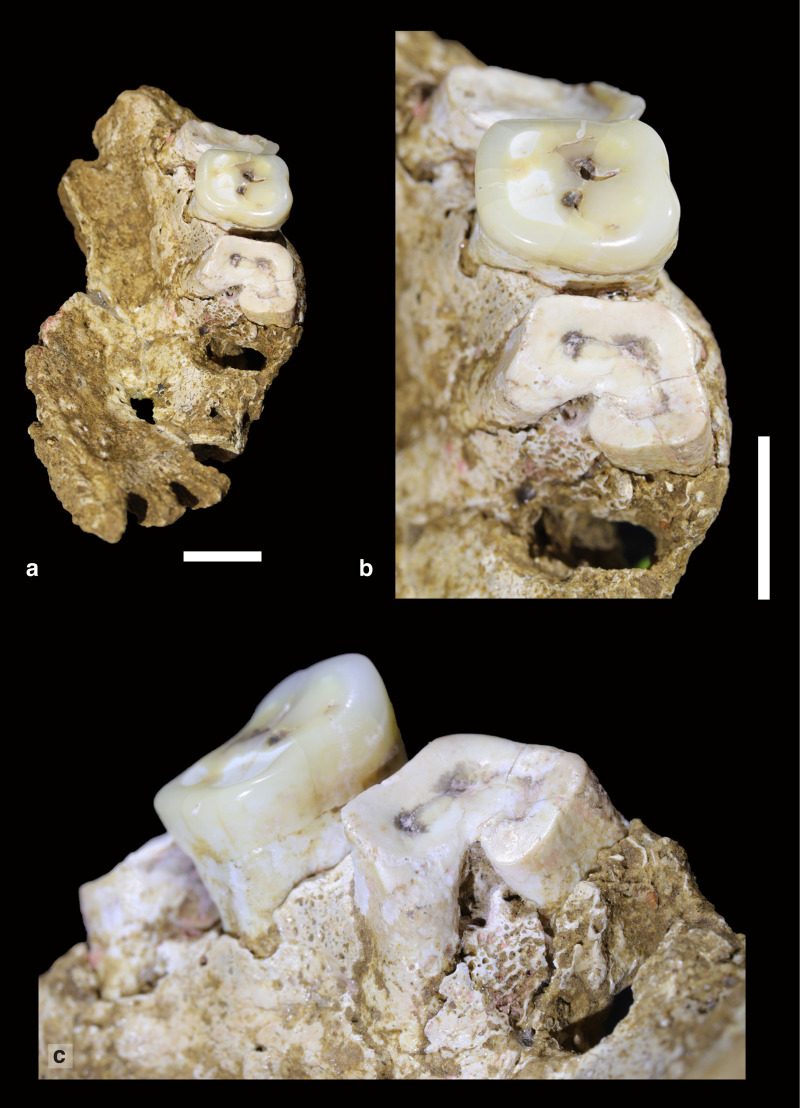
Dental pathologies in Maros-LBB-1a. (a) Inferior view of the right palate. Note the inflammation of the interproximal septa at all tooth sites, particularly marked at the sites of the premolar sockets, including the resorbed first premolar; (b) close-up of the inflammation of the interproximal septa at the second premolar site, showing also the rough interior surface of the socket. Note also the mesio-lingual to bucco-distal striation on the third molar; (c) close-up of the inflammation of the alveolar bone at the second premolar site, and slighter inflammation of the interproximal septa between the first and second molars. Scale bars are 10 mm.

We infer that this Late Pleistocene individual experienced poor oral health (Fig 9). The absence of any teeth other than the molars could be due to their loss after death, although none of them were recovered during excavation. Certainly, the modestly resorbed P^1^ alveolus (Fig 9) indicates that this tooth had been lost prior to the individual’s death, and the advanced dehiscences and rough socket surfaces at the incisor and canine sites (Fig 9) also suggest that these teeth had lost their anchoring before death. In addition, interproximal inflammation [43] was moderate to extensive at all tooth sites anterior of the M^2^, and all of the tooth sites show evidence for what we interpret as the effects of periodontal disease (Table 5). A level of antemortem tooth loss possibly as high as 50% of preserved sockets seems relatively high compared with other Pleistocene human fossil assemblages (see, e.g., [55]). At Grotte des Pigeons in Morocco, some 29% of post-canine teeth lost before death is recorded amongst biologically ‘old’ AMH adults of terminal Pleistocene antiquity, a phenomenon attributed to heavy occlusal attrition and cariogenic carbohydrates of a diet based on fermented pine nuts and acorns [56]. In Maros-LBB-1a, the only observable caries are pinhole-sized cavities in the anterior and central foveae of the M^2^. Hence, a closer comparison may be afforded by the Hoabinhian hunter-gatherer teeth from Gua Cha in the Peninsular Malaysia rainforests—a dramatic increase in dehiscences, interproximal inflammation and other periodontal disease with increased dental wear, but maintenance of a caries rate affecting only about 40% of teeth [40].

**Table 5:** Periodontal disease (after [4,4]) across the Maros-LBB-1a right maxilla.

Tooth site	Classification	Location	Classification
Central incisor	Marked resorption of the alveolar crest	Buccal	Reactive bone
Lateral incisor	Marked resorption of the alveolar crest	Buccal	Reactive bone
Canine	Marked resorption of the alveolar crest	Buccal	Reactive bone
First premolar	Marked resorption of the alveolar crest	Buccal and lingual	Marked resorption of the alveolar crest
Second premolar	Reactive bone	Lingual	Reactive bone
First molar	Irregular alveolar crest	Lingual	Irregular alveolar crest
Second molar	Pockets	Buccal	None
Third molar	Irregular alveolar crest	Buccal	Pockets

Maros-LBB-1a also exhibits a distinctive dental wear pattern (Figs 6 and 9). Only the M^2^, the occlusal surface of which projects below the other two extant molars, has a normal occlusal plane. The wear plane of the first molar slopes strongly upward from the distal to the mesial margin. A possible explanation for this unusual condition is that the first premolar alveolar surface had atrophied to the point where almost the entire root socket had been lost. Accordingly, during eating, the food bolus was masticated in a pronounced upward direction anteriorly from the first molar to the first premolar. On the other hand, the wear plane on the M^3^ slopes strongly upward mesially to distally. This would appear to be not due to mastication but instead the extensive use of this tooth as a tool; for instance, dragging palm fronds up the molar surface to produce twine. Possible evidence for this suggested paramasticatory (nondietary) dental use comes from the presence of a thin but clear striation running from the mesio-lingual to the bucco-distal surface of the tooth. Grooves on the anterior teeth resulting from twine production or similar activities are reported in the literature [57], but, in the case of Maros-LBB-1a, a striation rather than a groove is involved and the affected tooth is the most posterior in the dentition. Further assessment of the unusual tooth wear and oral pathology of the Leang Bulu Bettue palate is difficult for a damaged, isolated fragment.

### Teeth—Other observations

No signs of macroscopic enamel linear hypoplasia were observed on any of the extant molars of Maros-LBB-1a.

Observations on the root and crown morphology of Maros-LBB-1a are compatible with either a Sunda-Pacific (Southeast Asian/Micronesian/Polynesian) or a Sahul-Pacific (Australo-Melanesian) affinity, based on comparisons with the data in Scott and Turner [45]. The two-rooted first upper premolar (Fig 6) characterises ~40–60% of Sunda-Pacific and ~30–45% of Sahul-Pacific populations. The three-rooted upper second molar is found with ~50–80% of Sunda-Pacific and ~55–80% of Sahul-Pacific populations. The absence of enamel extension on the first upper molar (or any of the Maros-LBB-1a molars) characterises more than 80% of Sahul-Pacific populations and Micronesians, and is otherwise observed on ~65–80% of other Sunda-Pacific populations. Based on hypocone development recorded as ASU grade 4, the second upper molar can be clearly classified as four-cusped, as also recorded for 85–92% of Sunda-Pacific populations and consistently >90% of Sahul-Pacific populations.

Similarly, statistical analysis of dental morphology shows that the four closest populations to Gua Cha [58] are Sunda-Pacific (Polynesia, ’Early Southeast Asia’) or Sahul-Pacific (Aboriginal Australia, Melanesia).

### Cranial morphology

The anterior nasal spine is *absent* (Broca 1), and the right lower narial margin is represented by a thin line dividing the nasal and alveolar planes (*non-anthropine* of Larnach and Macintosh [47]). The lateral orbital margin of the malar is not preserved, but the medial (maxillary) section of the orbit appears to be trending towards a *rounded* orbital border. Medial to the orbit, the superior surface of the frontal process faces anterolaterally, suggesting at least modest anterior protrusion of the nasal bridge. Although the exact orientation in the original facial skeleton is unknown for the isolated frontal process, viewed laterally, the nasofrontal suture slopes anteriorly while the nasal margin follows a more vertical orientation. The surface immediately lateral to the lateral nasal margin forms a posteriorly ‘compressed’ or anteriorly faced, superoinferiorly elongated triangular area.

The shape of the palate is parabolic, and brachystaphylin or *broad* [41], with a breadth:length index of 93.8 (Fig 3). In sharp contrast to the modest anterior protrusion of the upper face suggested from the above structure, the subnasal part of the palate exhibits strong alveolar prognathism: in fact, subnasal prognathism is extreme (*large* of Larnach and Macintosh [47]).

The four characteristics italicised above reflect a morphology similar to that of Aboriginal Australians rather than to Europeans or East Asians [47]. As detailed in Table 6, based on the available comparative data, about 16% of Melanesians, 11% of Aboriginal Australians, and 5% of Island Southeast Asians equate with Maros-LBB-1a in presenting a broadly Australo-Melanesian morphology on all these four characters. Of the six Gua Cha crania with all of these characters intact, two of them (both Neolithic) also present a consistently Australo-Melanesian morphology [40].

**Table 6:** Recent (2000 BP and less) Indo-Pacific crania with Maros-LBB-1a cranial morphology^(a)^. ISEA = Island Southeast Asia.

Area covered	Observers	Females	Males	Combined
Melanesia				
New Guinea, New Britain, New Ireland, Solomons, Malekula, New Caledonia, "Melanesia"	Bulbeck^(b)^	3/29	19/107	22/136 = 16.2%
Australia				
Coastal New South Wales and Queensland	Larnach^(c)^	11/92	13/121	24/213 = 11.3%
ISEA				
Nicobar Islands, Malay Peninsula, Java, South Sulawesi, southeast Indonesia	Various^(d)^	3/40	4/110	7/150 = 4.7%

(a) *Large* subnasal prognathism, *absent* anterior nasal spine, *non-anthropine* narial margins and *rounded* orbital border. Only crania that could be scored for all four characters are included.

(b) Unpublished laboratory observations.

(c) Stanley Larnach papers, held at the State Library of South Australia.

(d) Unpublished laboratory observations by Johan Kamminga, David Bulbeck and Daniel Rayner.

Determining an affinity for the Gua Cha remains either with recent, local Island Southeast Asian populations or with Australo-Melanesian populations to the southwest proved exceptionally problematic [40], which invokes the caution recommended by Cunha and Ubelaker [59] in proposing ancestry based on conflicting or inadequate evidence. If this caution is appropriate for a sub-recent, well sampled series with several complete to semi-complete skulls, such as Gua Cha, it logically applies with even greater force to the highly fragmentary and much older Maros-LBB-1a remains.

## Discussion

The present scarcity of Late Pleistocene AMH skeletal remains in Wallacea means that our knowledge of the pattern and timing of the initial migration of our species into the region, and later interisland movements, is limited [2,60]. In prior decades, and continuing today, most attempts to model the earliest colonisation of the region by AMH have been based on the so-named ‘two-layer’ hypothesis or model. According to this concept, the first AMH to enter Wallacea at least 50 ka were Australo-Melanesians [61: p119]–direct lineal ancestors of modern-day Aboriginal Australians and Melanesians/Papuans [2,14]. The model holds that, once this founding population reached Sahul, it became cut-off and isolated in this continent until the middle Holocene period [62]. At this stage, direct contact with an as-yet unknown human population is indicated by the human-mediated dispersal of the dingo (*Canis dingo*) to mainland Australia, a wild canid that may have originally been introduced by Asian seafarers as a fully domesticated dog [63]. According to the two-layer model, the mainland East Asian affinity of modern people in the Philippines, Sulawesi, and islands to the west is due to the arrival ~5–4 ka of Neolithic farmers (‘Austronesians’) from a home base in southern China/Taiwan, and their absorption of the original Australo-Melanesians [64]. In this view, the Malayan-Papuan Line would mainly reflect the eastern limit of the immigrant farmers’ absorption of the pre-Austronesian inhabitants.

More complex models have also been proposed, with implications for a Pleistocene ancestry of the Malayan-Papuan Line. For instance, Karafet *et al*. [14] contend that following the initial peopling of Sahul by Australo-Melanesians Wallacea was the recipient of later migration events involving Late Pleistocene AMH colonists spreading out of Sunda from source populations with different genetic ancestry, and which never made it to Sahul. Claims have also been made for the presence of East Asian AMH populations in Sunda by around 40 ka [60; see also 65], as well as migrations of mainland East Asians into Wallacea during the LGM, a period of time when a combination of environmental changes affecting human subsistence and lower sea levels enhancing interisland visibility may have led to increased human population movements [2]. In a recent model, Curnoe *et al*. [60] propose that: 1) AMH of African origin reached southern China by 80 ka [66] and from there migrated into northern Sunda (present-day Borneo) and across the Wallace Line to Sulawesi, and possibly to Maluku, but did not extend their range into Sahul. Based on Curnoe *et al’s* [60] reanalysis of the Niah Cave ‘Deep Skull’ (~37 ka), the oldest *H*. *sapiens* fossil known from Borneo, it is proposed that this early AMH group had a close morphological affinity with present-day mainland East Asians; and 2) a separate wave of AMH colonists of Australo-Melanesian affinity migrated eastward along the southernmost edge of Sunda and dispersed along the southern route across Wallacea into Sahul. According to this model, contrary to the two-layer hypothesis there was no major turnover in biological populations associated with the transition from foraging to farming in Island Southeast Asia: ‘Instead, it seems more likely that Austronesian speakers from Taiwan and island Southeast Asia share a common origin going back to the Late Pleistocene with only a limited signal of the “Out-of-Taiwan” expansion during the Neolithic period’ [60: p15].

As noted, the human skeletal remains from Leang Bulu Bettue dated to ~25–16 ka are the first fossil evidence of hominins recovered thus far from Pleistocene Sulawesi, a key island in our understanding of the pattern of AMH colonisation of Wallacea and Sahul. For what it is worth, these highly fragmentary materials present characters that would be consistent with either an Australo-Melanesian or an Island Southeast Asian affinity, and so this specimen cannot be considered as providing empirical support for either the two-layer model or any of its contenders.

In summary, the two-layer hypothesis holds that the original founding wave of Australo-Melanesians that colonised Wallacea in the Late Pleistocene gave rise to localised island populations that remained isolated genetically and culturally over tens of thousands of years until the arrival of East Asian farmers (‘Austronesians’) in the middle Holocene. It has also been proposed that, following the initial peopling of Sahul by Australo-Melanesians, Wallacea was the recipient of further Late Pleistocene migration events involving AMH spreading into the region from sources in mainland East Asia and intermingling with established Australo-Melanesian populations. Presently, using only the fossil record to go by, it is difficult to test these scenarios on a regional scale and across the known time span of Late Pleistocene occupation of Wallacea by AMH, owing to the scarcity of human skeletal materials in the region. Genomic analyses of living people and ancient DNA sequences are advancing our knowledge of early human migrations and population histories in the region (e.g., Lipson *et al*. [67]; McColl *et al*. [68]); thus far, however, there are no ancient human genetic materials from Wallacea (including Maros-LBB-1a) and hence our understanding of how the modern pattern of human genetic diversity in the region arose, including the origins of the Malayan-Papuan Line, is poorly developed.

Finally, it is worth us highlighting that the Leang Bulu Bettue individual possibly belonged to the population responsible for one of the world’s oldest known rock art traditions. As mentioned above, dated parietal art in the surrounding Maros-Pangkep karsts spans the time period from at least 45.5 ka until the LGM. The human remains came from Layer 4a, a rich archaeological horizon that yielded diverse and regionally unique evidence for portable art, as well as personal ornaments and pigment use [10]. The former includes small rocks engraved with abstract markings, and, in two cases, figurative imagery [33]. These art objects and ornaments were created long after the most likely initial period of settlement of Wallacea by AMH (~70–50 ka). However, coupled with the U-series rock art dates from Maros-Pangkep, they suggest that a sophisticated artistic culture existed in South Sulawesi from at least 45.5 ka until around the time of the LGM. Similarly, one Tron Bon Lei burial (from Test Pit B) was interred with elaborately crafted shell fishhooks [11], highlighting the importance of symbols in the lives of Late Pleistocene AMH in Alor and perhaps within the cultural worlds of ‘ice age’ communities in Wallacea generally [10,69].

## Conclusion

There are many unknowns in our understanding of the early history of our species in Wallacea. Given the dearth of fossil data, the recovery of any new AMH skeletal element from Pleistocene Wallacea, even highly fragmentary remains like the right maxilla Maros-LBB-1a from Leang Bulu Bettue, is of value; at least to the extent of demonstrating the presence of early *H*. *sapiens* in a region that may have been host to multiple species of archaic hominins. The specimen also has the advantage of being securely dated by a variety of chronometric techniques to ~25 to 16 ka. The first modern humans to reach Sulawesi produced some of the oldest known dated rock art [13,30], yet little is known about the origin and cultural lives of these Late Pleistocene hunter-gatherers. Maros-LBB-1a provides us with the first direct fossil insight into the identity of these ancient foragers, and its unusual tooth wear and oral pathology offer tantalising hints on how they adapted to their rainforest environment. It is clear that much more basic fieldwork remains to be done, however, in order to unravel the cultural and biological history of early AMH in this Wallacean island.

## Supporting information

S1 Fig 3D Photoscan model of Maros-LBB-1a. Credit: D.P. McGahan.
(PDF)
